# Cross-sectional associations between accelerometry-measured physical activity, left atrial size, and indices of left ventricular diastolic dysfunction: The Tromsø Study

**DOI:** 10.1016/j.pmedr.2020.101290

**Published:** 2020-12-31

**Authors:** Kim Arne Heitmann, Maja-Lisa Løchen, Laila A. Hopstock, Michael Stylidis, Boye Welde, Henrik Schirmer, Bente Morseth

**Affiliations:** a School of Sport Sciences, UiT The Arctic University of Norway, Tromsø, Norway; b Centre for Clinical Research and Education, University Hospital of North Norway, Tromsø, Norway; c Department of Community Medicine, UiT The Arctic University of Norway, Tromsø, Norway; d Department of Cardiology, Akershus University Hospital, Lørenskog, Norway; e Institute of Clinical Medicine, University of Oslo, Oslo, Norway; f Department of Clinical Medicine, UiT The Arctic University of Norway, Tromsø, Norway

**Keywords:** Heart, Exercise, Ageing, Echocardiography, Public health

## Abstract

Whereas left atrial (LA) enlargement is an independent predictor for adverse cardiovascular events and all-cause mortality, this is regarded a physiological adaption of exercise. Paradoxically, LA size in athletes may overlap the enlargement observed in patients with cardiac pathology. Current knowledge is mainly derived from studies of athletes, and little is known about cardiac adaptations to physical activity (PA) in the general population. We explored the association between objectively measured PA and LA volume index (LAVi), and between LAVi enlargement and indices of diastolic dysfunction stratified by PA-level.

Our study included 1573 participants from the population-based Tromsø Study (2015–16). PA was assessed with an ActiGraph wGT3X-BT accelerometer. Echocardiography was performed according to current guidelines. The associations between PA and LAVi, and between LAVi enlargement and indices of diastolic dysfunction were estimated by univariable and multivariable linear regression analyses, adjusted for sex, age, and cardiovascular risk factors.

Our multiple adjusted analyses showed significant linear associations between PA and LAVi in ages < 70 years, and between PA and LAVi in participants with normal diastolic function. No associations were seen in ages ≥ 70 years or for participants with abnormal diastolic function. In those 40–54 years, the most active participants had larger LAVi (4.45 mL/m^2^, p = 0.016) than the least active. LAVi enlargement was only associated with indices of diastolic dysfunction in the most inactive participants.

In conclusion, higher levels of PA associate with greater LAVi in participants < 70 years with normal diastolic function. LAVi enlargement is only associated with diastolic dysfunction in the most inactive participants.

## Highlights

• Examination of how objectively PA and LAVi associate in a general population represents novelty.
• PA is associated with greater LA size in participants <70 years with normal diastolic function.
• LA enlargement is only associated with diastolic dysfunction in the most inactive participants.
• We suggest that LA enlargement in active individuals is not an expression of cardiac dysfunction.

## 1 Introduction

Increasing age is associated with adverse structural and functional changes in the heart, such as left atrial (LA) enlargement (Obas and Vasan, 2018). LA enlargement is an independent predictor for adverse cardiovascular disease (CVD) (Tiwari et al., 2015) and all-cause mortality (Stylidis et al., 2019). Furthermore, LA enlargement is a marker of the severity and chronicity of left ventricular (LV) diastolic dysfunction (Lang et al., 2015), and an important prevention target due to increased risk for heart failure (HF) and CVD events (Gajardo and Llancaqueo, 2019).

Health authorities recommend physical activity (PA) for health promotion and disease prevention, such as CVD and all-cause mortality (Piepoli et al., 2016, Bull et al., 2020, The 2018 Physical Activity Guidelines Advisory Committee, 2018), and improved diastolic and systolic function (Hegde et al., 2016). However, little is known about cardiac adaptations to PA in the general adult and elderly population (Joseph et al., 2019); current knowledge is mainly derived from studies of athletes (Hegde et al., 2016). Furthermore, the associations between PA and health outcomes are primarily based on studies using self-reported PA (The 2018 Physical Activity Guidelines Advisory Committee, 2018), which are prone to recall and social desirability biases (Prince et al., 2008).

Exercise-induced cardiac remodelling is considered a benign physiological adaption of athletic training, characterized by chamber enlargements and increased LV wall thickness (D’Ascenzi et al., 2020). Furthermore, a meta-analysis comprising over 7000 elite athletes, reported that athletes had 30% greater LA size compared with sedentary controls, when correcting for body surface area (Iskandar et al., 2015). Paradoxically, LA enlargement in athletes may overlap the LA dilation observed in patients with cardiac pathology, and thus represent a diagnostic challenge for clinicians (D'Ascenzi et al., 2018). The need for future research to investigate the possibility of increased risk associated with high amounts of PA is highlighted (The 2018 Physical Activity Guidelines Advisory Committee, 2018), as the upper and lower limits of PA to exert a beneficial effect is unknown (Piepoli et al., 2016).

We aimed to provide new knowledge on cardiac adaptations to PA in the general adult and elderly population. Our main objectives were to: a) explore the association between objectively measured PA and LA size, and b) investigate if LA enlargement is adversely associated with indices of diastolic dysfunction when correcting for PA. We hypothesized that LA size is associated with PA, and that LA enlargement is not adversely associated with indices of diastolic dysfunction in the most active participants.

## 2 Methods

### 2.1 Study population

The Tromsø Study (Jacobsen et al., 2012) is a single center population-based cohort study with seven repeated health surveys of the population of the Tromsø municipality, Northern Norway. This cross-sectional study includes participants from the 7th survey (2015–16) (The Tromsø Study, 2016). All inhabitants ≥ 40 years were invited (n = 32591), with 21,083 men and women participating (65% attendance). The participants completed questionnaires and underwent biological sampling and clinical examinations. Of these, a sample of 8346 participants (randomly assigned or attendees of dual-energy x-ray absorptiometry, echocardiography and eye examination at the 6th survey in 2007–08) participated in a second visit with extended examinations. Among these, 6125 participants provided valid data on accelerometry, and 2340 participants had valid echocardiographic data. In total, 1651 participants had valid data on both echocardiography and accelerometery. Due to missing data for one or more covariates, 78 participants were excluded (height n = 2, systolic blood pressure n = 5, diabetes n = 44, smoking n = 17, low-density lipoprotein (LDL) cholesterol n = 10), leaving an analytical sample of 1573 participants (Table 1).

**Table 1:** Descriptive characteristics of the study population by age groups: The Tromsø Study 2015–16.

**Variable**	**40**–**54 years (n = 305)**	**55**–**69 years (n = 729)**	**≥70 years (n = 539)**
Age, years	47.7 (4.4)	63.3 (4.0)	75.7 (4.0)
Gender, % (n) female	59.3 (181)	53.1 (387)	45.8 (247)
Body mass index, kg/m^2^	27.1 (5.0)	27.0 (4.2)	27.2 (4.1)
Systolic blood pressure, mmHg	121.9 (16.4)	132.9 (18.8)	143.2 (20.7)
Diastolic blood pressure, mmHg	74.3 (10.3)	76.0 (9.9)	74.3 (9.9)
LDL cholesterol, mmol/L	3.5 (0.9)	3.7 (1.0)	3.3 (1.1)
Smoking, % (n)^a^	55.7 (170)	64.1 (467)	61.4 (331)
Hypertension, untreated, % (n)^b^	17.0 (52)	36.1 (263)	54.2 (292)
Hypertension, controlled, % (n)^c^	4.9 (15)	15.9 (115)	22.6 (121)
Myocardial infarction, % (n)	0 (0)	4.8 (35)	13.3 (70)
Stroke, % (n)	0 (0)	2.6 (19)	6.6 (35)
Diabetes, % (n)	3.6 (11)	5.9 (43)	10.2 (55)
**Echocardiography**			
LA volume index, mL/m^2^	32.0 (9.0)	32.6 (10.9)	37.8 (14.7)
LV mass index male, g/h^2.7^	41.7 (11.3)	46.3 (13.9)	51.8 (18.9)
LV mass index female, g/h^2.7^	36.2 (9.8)	41.2 (12.5)	47.5 (18.6)
e’ velocity, lateral, cm/s	12.7 (3.0)	9.6 (2.4)	8.0 (2.4)
e’ velocity, septal, cm/s	9.8 (2.2)	7.4 (2.1)	6.2 (2.1)
E/e’ratio	7.5 (2.0)	8.9 (3.4)	11.0 (6.7)
Tricuspid regurgitation velocity, m/s	1.4 (0.7)	1.5 (0.8)	1.7 (0.8)
Ejection fraction < 40%, %	5.8 (15)	6.9 (44)	12.4 (57)
**Physical activity**			
Mean wear time all valid days, hours	118.0 (15.4)	118.4 (16.1)	113.0 (17.4)
Total PA, CPM	611.6 (174.1)	550.9 (170.4)	449.9 (159.6)
PA-quartile 1, % (n)	9.5 (29)	18.9 (138)	41.9 (226)
PA-quartile 2, % (n)	23.9 (73)	25.4 (185)	25.2 (136)
PA-quartile 3, % (n)	25.2 (77)	27.7 (202)	21.0 (113)
PA-quartile 4, % (n)	41.3 (126)	28.0 (204)	11.9 (64)
MVPA, min/day	53.5 (30.5)	44.5 (29.9)	26.8 (24.9)
Steps, steps/day	8214.5 (2714.3)	7431.7 (2889.9)	5231.3 (2459.3)

LDL: low-density lipoprotein, LA: left atrium, LV: left ventricle, E/e’ratio: ratio of mitral peak velocity of early filling (E) to early diastolic mitral annular velocity (e'), CPM: counts per minute, PA: physical activity, MVPA: moderate-to-vigorous physical activity.

Numbers are mean ± standard deviation or percentage and n.

a Smoking: current and previous.

b Hypertension, untreated: systolic blood pressure ≥ 140 mmHg and/or diastolic blood pressure ≥ 90 mmHg and no self-reported use of antihypertensives.

c Hypertension, controlled: systolic blood pressure < 140 mmHg and diastolic blood pressure < 90 mmHg, and self-reported use of antihypertensives.

The present study was approved by the Regional Committee for Medical and Health Research Ethics (2017/1973/REK Nord) and carried out in accordance with the Declaration of Helsinki. All participants signed written informed consent.

### 2.2 Assessment of PA

PA was assessed objectively in three axes with an accelerometer (wGT3X-BT, ActiGraph LLC, Pensacola, FL, USA) placed on the participants’ right hip. The participants were instructed to perform their daily activities as usual, and to wear the accelerometer day and night for eight consecutive days, except during water activities. Valid measurements were defined as wear-time ≥ 10 h/day for ≥ 4 days. Details of the data collection and accelerometer data processing procedures are described elsewhere (Sagelv et al., 2019). The accelerations are expressed as average counts per minute (CPM), and categorized into moderate-to-vigorous PA (MVPA, ≥ 2690 CPM) based on validated cut-points (Sasaki et al., 2011). Steps were collected from acceleration on the vertical axis. The participants’ CPM were furthermore divided into PA-quartiles, with the least active quartile 1 as reference: quartile 1 = ≤406 CPM, quartile 2 = 407–514 CPM, quartile 3 = 515–634 CPM and quartile 4 = ≥635 CPM.

### 2.3 Data collection

Data from questionnaires, physical examinations and blood samples were used for the following covariates: Smoking (currently/previously, never), diabetes (currently/previously, no), use of antihypertensives (currently, previously/never), myocardial infarction (previously, no) and stroke (previously, no). Body mass index (BMI) was calculated as weight (kg) divided by height squared (m^2^). Blood pressure was recorded three times with 1 min interval after 2 min rest, using an automatic device (Dinamap ProCare 300 monitor, GE Healthcare, Norway), the average from reading two and three was used. Blood samples were analysed for LDL cholesterol at the Department of Laboratory Medicine, University Hospital of North Norway.

### 2.4 Cardiac structure and function

Echocardiography was performed by a qualified sonographer between August 2015 and October 2016, using a GE Vivid E9 (GE Medical, Horten, Norway) ultrasound scanner. Offline image readings and analyses were performed by co-author M.S. with the commercially available EchoPac software (Ver. 113, GE Vingmed Ultrasound AS, Horten, Norway). Echocardiographic assessment was performed with the use of standard imaging planes in the left lateral decubitus position according to the joint American and European guidelines (Lang et al., 2015, Nagueh et al., 2016). The echocardiographic assessment was performed on 3–5 consecutive cardiac cycles, and the average was used in the analysis.

LA volume was measured at the end of the LV systole and calculated using the Simpson’s biplane method from the apical four- and two chamber views. The LA volume was indexed to body surface area (LAVi) using the Mosteller formula (Mosteller, 1987). Mitral annular e’ velocity was measured in apical four-chamber view with pulsed-wave tissue Doppler in both lateral and septal basal regions. Average E/e’ratio was calculated by peak E-wave velocity divided by annular average e’ velocity both collected in apical four-chamber view. Tricuspid regurgitation velocity (TRV) was measured as peak modal velocity from the right ventricle to the right atrium during systole, using apical four-chamber view with continuous wave Doppler. LAVi, E/e’ratio, e’ velocity and TRV were used to consider diastolic function according to the guidelines (Nagueh et al., 2016). In our analysis, diastolic function was considered as: a) normal if < 50% of the variables were above the cut-offs, or b) abnormal if ≥ 50% of the variables were above the cut-offs.

LV myocardial mass was calculated according to the cube formula (Lang et al., 2015), and further indexed to height by raising height to the power of 2.7 (Williams et al., 2018). LV volume was calculated at the end of diastole, using the Simpson’s biplane method from the apical four- and two chamber views. Ejection fraction was calculated from the end-diastolic and end-systolic estimates of LV volume, and individuals with ejection fraction < 40% were considered as having HF (Ponikowski et al., 2016). Severity of the mitral and aortic stenosis was assessed by measuring mitral valve area and aortic flow mean pressure gradient, respectively. Mitral regurgitation severity grade was obtained by qualitative estimation of the color Doppler flow jet area. Pressure half-time of regurgitant aortic flow as well as central jet width were used for assessment of the severity of the aortic regurgitation (Zoghbi et al., 2017).

An intra- and inter-observer study was performed on the echocardiography data (Stylidis et al., 2020). Intra-class correlation coefficients on Doppler indices and linear measurements were 0.90–0.99 in the intra-observer study, and 0.84–0.98 in the inter-observer study.

### 2.5 Statistical methods

The associations between the PA measures and LAVi, and between LAVi enlargement and indices of diastolic function were estimated by univariable and multivariable linear regression analyses. The associations are presented as unstandardized coefficients with 95% confidence intervals (CI). Coefficients for MVPA are presented for every 10 min increase per day (MVPA per 10 min/day), and steps are presented for every 1000 steps per day increase (steps per 1000/day). The multivariable model (Model 2) was adjusted for sex, age, and CVD risk factors (BMI, systolic blood pressure, diabetes, smoking, and LDL cholesterol). In a sub-analysis, we used an extended multivariable model (Model 3), which was adjusted for Model 2 in addition to myocardial infarction, HF, mitral regurgitation, mitral stenosis, aortic regurgitation, and aortic valve flow mean pressure gradient.

In the multivariable model with the PA measures as exposure and LAVi as outcome, we first included stepwise interactions between PA*age, PA*sex, PA*hypertension, PA*BMI, PA* diastolic function, PA*CVD, and PA*LV mass index. We then included only the significant interaction terms (PA*age, and PA*diastolic function) in the multivariable model. We stratified our main analyses by three age groups and diastolic function, as the interactions between PA*age, and PA*diastolic function were significant. All statistical analyses were performed using SPSS version 25 (SPSS Inc., IL, USA), with the alpha level set to 0.05.

## 3 Results

Descriptive characteristics from questionnaires, biological samplings, clinical examinations, and assessment of PA are given for the participants stratified by age groups in Table 1. The mean age was 64.5 (SD 10.7, range 40–84) years and mean BMI was 27.1 (SD 4.3) kg/m^2^. The proportion of men increased with increasing age (p-trend < 0.001), and all measures of PA decreased with increasing age (p-trend < 0.001).

### 3.1 Association between PA and LAVi

In the overall sample, there was a significant trend towards increasing LAVi with increasing PA-quartiles in the fully adjusted Model 2 (p-trend = 0.017, Table S1). Participants with the highest PA level (quartile 4) had larger LAVi (2.31 mL/m^2^, p = 0.015) than participants in quartile 1. LAVi increased non-significantly with increasing MVPA (p = 0.176) and steps (p = 0.184).

**Fig. 1:**
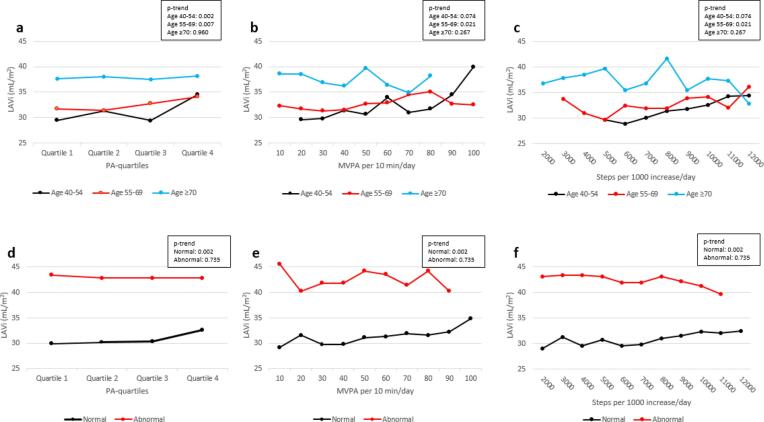
**a-c** show adjusted mean LAVi stratified by age and PA, and Fig. 1**d-f** show adjusted mean LAVi stratified by LV diastolic function and PA. Fig. 1a and 1d show mean LAVi for the PA-quartiles, where quartile 1 is least active and quartile 4 most active. Fig. 1b and 1e show mean LAVi for every 10 min increase in MVPA per day, and Fig. 1c and 1f show mean LAVi for every 1000 steps per day increase. Mean values were adjusted for sex, BMI, systolic blood pressure, diabetes, smoking, and LDL cholesterol. LAVi: left atrial volume index, PA: physical activity, MVPA: moderate-to-vigorous physical activity, BMI: body mass index, LDL: low-density lipoprotein. The Tromsø Study 2015–16.

**Fig. 2:**
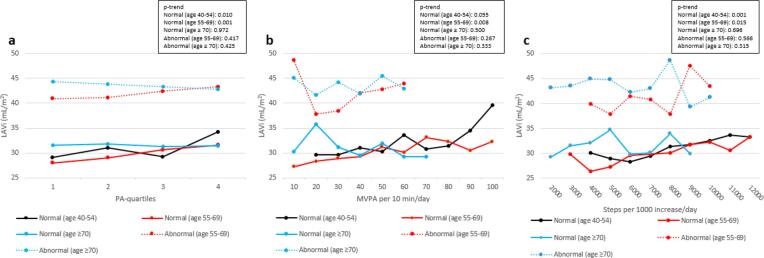
Adjusted mean LAVi stratified by LV diastolic function, age and PA. Fig. 2a show mean LAVi for the PA-quartiles, where quartile 1 is least active and quartile 4 most active. Fig. 2b show mean LAVi for every 10 min increase in MVPA per day, and Fig. 2c show mean LAVi for every 1000 steps per day increase. Mean values were adjusted for PA level, sex, BMI, systolic blood pressure, diabetes, smoking, and LDL cholesterol. LAVi: left atrial volume index, PA: physical activity, MVPA: moderate-to-vigorous physical activity, BMI: body mass index, LDL: low-density lipoprotein. The Tromsø Study 2015–16.

We found significant interactions between PA*age, and between PA*diastolic function in the association between PA and LAVi. The association between PA and LAVi showed a positive trend in age groups < 70 years (Fig. 1a-c), whereas no significant association between PA and LAVI among those aged ≥ 70. Furthermore, the association between PA and LAVi showed a positive trend in participants with normal diastolic function (Fig. 1d-f), but no significant association between PA and LAVi in participants with abnormal diastolic function. When both the interactions between PA*age, and PA*diastolic function were added to the multiple adjusted Model 2 (Table S2), interactions between PA*age, and PA*diastolic function remained significant (p < 0.05). Therefore, further analyses were stratified by age and diastolic function. The association between PA and LAVi showed a positive trend in participants < 70 years with normal diastolic function, whereas no significant association was observed in participants ≥ 70 years or in participants with abnormal diastolic function (Fig. 2).

When stratified by age (Table 2), both models showed positive associations between PA and LAVi in the age groups < 70 years. In those aged 40–54 years, LAVi increased by 0.62 mL/m^2^ for every 1000 steps per day increase (p = 0.002). In addition, there was a significant trend showing increased LAVi by increasing PA-quartiles (p-trend = 0.009). LAVi was significantly larger (4.45 mL/m^2^, p = 0.016) in participants in PA-quartile 4 vs. quartile 1. In participants aged 55–69 years, LAVi increased by 0.32 mL/m^2^ for every 10 min increase in MVPA per day (p = 0.021). There was also a significant trend showing increased LAVi by increasing PA-quartiles (p-trend = 0.007). LAVi was significantly larger (2.93 mL/m^2^, p = 0.020) in participants in PA-quartile 4 vs. quartile 1. There were no significant associations between PA and LAVi in participants aged ≥ 70 years.

**Table 2:** Associations between physical activity and left atrial volume index by age (unstandardized coefficients ± 95% CI). The Tromsø Study 2015–16.

	n	LAVi mL/m^2^ (SD)	Model 1 β (95% CI)	Model 2 β (95% CI)
**Age 40**–**54**				
CPM	297	32.0 (9.0)		
PA-quartile 1	28	29.5 (6.6)	0.00 (Ref.)	0.00 (Ref.)
PA-quartile 2	71	31.3 (11.1)	1.81 (-2.02, 5.65)	1.90 (-1.93, 5.77)
PA-quartile 3	75	29.4 (7.9)	−0.09 (-3.89, 3.71)	0.05 (-3.76, 3.87)
PA-quartile 4	123	34.5 (8.1)	5.05 (1.46, 8.64)	4.45 (0.82, 8.08)
p-trend			0.001	0.009
MVPA per 10 min/day	297	32.0 (9.0)	0.41 (0.08, 0.74)	0.31 (-0.03, 0.65)
p-trend			0.015	0.074
Steps per 1000/day	297	32.0 (9.0)	0.77 (0.40, 1.13)	0.62 (0.23, 1.00)
p-trend			<0.001	0.002
**Age 55**–**69**				
CPM	714	32.6 (10.9)		
PA-quartile 1	131	31.7 (12.3)	0.00 (Ref.)	0.00 (Ref.)
PA-quartile 2	184	31.4 (10.0)	−0.36 (-2.81, 2.09)	0.01 (-2.45, 2.47)
PA-quartile 3	199	32.8 (10.6)	1.04 (-1.36, 3.45)	1.30 (-1.12, 3.72)
PA-quartile 4	200	34.1 (10.9)	2.33 (-0.07, 4.74)	2.93 (0.46, 5.41)
p-trend			0.018	0.007
MVPA per 10 min/day	714	32.6 (10.9)	0.33 (0.07, 0.60)	0.32 (0.05, 0.59)
p-trend			0.015	0.021
Steps per 1000/day	714	32.6 (10.9)	0.23 (-0.05, 0.51)	0.26 (-0.03, 0.55)
p-trend			0.110	0.081
**Age ≥ 70**				
CPM	531	37.8 (14.7)		
PA-quartile 1	224	37.7 (15.4)	0.00 (Ref.)	0.00 (Ref.)
PA-quartile 2	134	38.0 (14.6)	0.34 (-2.83, 3.51)	0.08 (-3.14, 3.29)
PA-quartile 3	110	37.5 (15.1)	−0.19 (-3.57, 3.19)	−0.23 (-3.67, 3.22)
PA-quartile 4	63	38.1 (11.2)	0.39 (-3.75, 4.53)	0.35 (-3.89, 4.58)
p-trend			0.938	0.960
MVPA per 10 min/day	531	37.8 (14.7)	−0.26 (-0.76, 0.24)	−0.30 (-0.82, 0.23)
p-trend			0.312	0.267
Steps per 1000/day	531	37.8 (14.7)	−0.21 (-0.72, 0.30)	−0.24 (-0.78, 0.29)
p-trend			0.425	0.374

LAVi: left atrial volume index, SD: standard deviation, CI: confidence interval, Ref.: reference, CPM: counts per minute, PA: physical activity, MVPA: moderate-to-vigorous physical activity, BMI: body mass index, LDL: low-density lipoprotein.

Model 1 was unadjusted. Model 2 was adjusted for sex, BMI, systolic blood pressure, diabetes, smoking, and LDL cholesterol.

Associations between PA and LAVi stratified by diastolic function showed increased LAVi by increasing PA-quartiles in participants with normal diastolic function (p-trend = 0.002) (Table 3). In participants with normal diastolic function, LAVi was significantly larger (2.77 mL/m^2^, p = 0.002) in participants in PA-quartile 4 vs. quartile 1. Furthermore, LAVi increased by 0.22 mL/m^2^ for every 10 min increase in MVPA per day (p = 0.033), and by 0.29 mL/m^2^ for every 1000 steps per day increase (p = 0.009). There were no significant associations between PA and LAVi in participants with abnormal diastolic function.

**Table 3:** Associations between physical activity and left atrial volume index by left ventricular diastolic function (unstandardized coefficients ± 95% CI). The Tromsø Study 2015–16.

	n	LAVi mL/m^2^ (SD)	Model 1 β (95% CI)	Model 2 β (95% CI)
**Normal diastolic function**^a^				
CPM	1105	30.8 (9.4)		
PA-quartile 1	236	29.9 (10.0)	0.00 (Ref.)	0.00 (Ref.)
PA-quartile 2	281	30.2 (10.2)	0.30 (-1.32, 1.93)	0.42 (-1.25, 2.08)
PA-quartile 3	288	30.4 (9.0)	0.50 (-1.11, 2.12)	0.64 (-1.03, 2.31)
PA-quartile 4	300	32.6 (8.3)	2.77 (1.17, 4.37)	2.77 (1.02, 4.51)
p-trend			0.001	0.002
MVPA per 10 min/day	1105	30.8 (9.4)	0.25 (0.06, 0.43)	0.22 (0.02, 0.42)
p-trend			0.009	0.033
Steps per 1000/day	1105	30.8 (9.4)	0.30 (0.11, 0.48)	0.29 (0.07, 0.50)
p-trend			0.002	0.009
**Abnormal diastolic function**^b^				
CPM	437	43.0 (14.3)		
PA-quartile 1	147	43.4 (16.2)	0.00 (Ref.)	0.00 (Ref.)
PA-quartile 2	108	42.8 (12.9)	−0.64 (-4.22, 2.94)	−0.66 (-4.29, 2.97)
PA-quartile 3	96	42.8 (14.7)	−0.59 (-4.29, 3.12)	−0.36 (-4.16, 3.44)
PA-quartile 4	86	42.8 (12.2)	−0.67 (-4.50, 3.17)	−0.79 (-4.85, 3.27)
p-trend			0.721	0.735
MVPA per 10 min/day	437	43.0 (14.3)	0.02 (-0.44, 0.49)	−0.02 (-0.52, 0.48)
p-trend			0.925	0.928
Steps per 1000/day	437	43.0 (14.3)	−0.14 (-0.60, 0.33)	−0.18 (-0.70, 0.34)
p-trend			0.567	0.490

LAVi: left atrial volume index, SD: standard deviation, CI: confidence interval, Ref.: reference, CPM: counts per minute, PA: physical activity, MVPA: moderate-to-vigorous physical activity, BMI: body mass index, LDL: low-density lipoprotein.

Model 1 was unadjusted. Model 2 was adjusted for age, sex, BMI, systolic blood pressure, diabetes, smoking, and LDL cholesterol.

a Normal diastolic function = <50% positive variables.

b Abnormal diastolic function = ≥50% positive variables.

In general, associations between PA-quartiles and LAVi stratified by hypertension, CVD, LV mass index or sex (Table S3-S6) showed rather similar results as when stratifying for diastolic function. No associations were observed when stratifying by BMI (Table S7). Furthermore, we explored the associations between PA and LAVi using an extended model (Model 3) adjusted for myocardial infarction, HF and valvular disorders in addition to Model 2. In general, we observed similar associations between PA and LAVi as in Model 2, although, some of the associations were slightly stronger in Model 3. As for Model 2, we observed associations between PA and LAVi in participants < 70 years (Table S8), and in participants with normal diastolic function (Table S9). There were no associations between PA and LAVi in participants ≥ 70 years or with abnormal diastolic function.

### 3.2 Association between LAVi enlargement and indices of diastolic dysfunction

LAVi enlargement was associated with unfavorable increase in E/e’ratio (0.24, p < 0.001) and TRV (0.02 m/s, p < 0.001) for every mL/m^2^ increase in the most inactive participants, whereas no associations were seen in higher PA-quartiles (Table S10).

### 3.3 Sensitivity analysis

When excluding participants with atrial fibrillation (AF, n = 44), we observed similar associations between PA and LAVi, and between LAVi enlargement and indices of diastolic dysfunction as the main results. Therefore, we included participants with AF in our analysis.

## 4 Discussion

To the best of our knowledge, this is the first study that examined the association between objectively measured PA and LAVi in the general adult and elderly population. Our main findings are that higher levels of PA are associated with greater LAVi, but only in participants < 70 years with normal diastolic function, and that LAVi enlargement is associated with indices of diastolic dysfunction only in the most inactive participants. Our results indicate that LAVi enlargement in physically active individuals is not an expression of cardiac dysfunction.

### 4.1 PA and LA size

When stratified by age and diastolic function in the same multivariable model, we observed significant trends between PA and LAVi in age groups < 70 years with normal diastolic function only. These findings suggest that the relationship between higher age and increased LA size is mainly explained by diastolic function, as there was no difference between age groups when excluding those with diastolic dysfunction.

Similar to our results, Joseph et al. (2019) reported that PA had greater impact on cardiac alterations in younger than older subjects in the general population. They reported that higher levels of PA improved both systolic and diastolic function (evaluated by longitudinal displacement, e’, and myocardial performance index) and lowered resting heart rate in participants ≤ 65 years, whereas there was no effect of PA in those > 65 years. Furthermore, the authors suggested that there is a cut-off point in age, where the beneficial effects of PA ceases.

The positive association between PA and LAVi observed in our study is widely supported by previous studies on athletes (Iskandar et al., 2015, Pelliccia et al., 2005). In addition, the association between exercise and LAVi has been demonstrated in adolescent endurance athletes (Hoogsteen et al., 2003). In the general adult population, the association between cardiorespiratory fitness and LAVi (Letnes et al., 2020), and the association between objectively measured PA and LA dimension has been demonstrated (Andersson et al., 2015). Furthermore, the relationship between exercise and LAVi has also been confirmed in a randomized controlled trial with middle-aged adults (Opondo et al., 2018). In contrast to our results, Bjerring et al. (2018) found no association between exercise and LAVi although they observed other associations between exercise and cardiac remodelling in preadolescent athletes. The lack of significant association between exercise and LAVi may be due to the participants’ young age, and because atrial remodelling depends on years of training (D'Andrea et al., 2010).

Enlarged LAVi is considered a normal physiological adaptation to exercise in athletes, and it is well documented that the cardiac chambers increase by exercise (Iskandar et al., 2015, Pelliccia et al., 2005). Enlarged chambers and the accompanying capability to generate a large stroke volume are the main alterations by exercise-induced cardiac remodelling (Weiner and Baggish, 2012), and a large stroke volume is essential for athletic endurance and performance (Bassett and Howley, 2000). Our results indicate that the exercise-induced cardiac adaptations start at lower levels of PA than the typical training load of an athlete, and that exercise-induced LA remodelling also affects the general and elderly population.

In athletes, it has been demonstrated that LA enlargement is not necessarily associated with LA dysfunction (D'Ascenzi et al., 2011). In fact, supernormal LV diastolic function has been documented in athletes with LA enlargement (D'Ascenzi et al., 2011). These athletes typically have normal reservoir function and reduced LA active contraction (D'Ascenzi et al., 2018). Reduced LA active contraction represents a more efficient filling of the heart, due to larger contribution from passive hemodynamic instead of active contractions (Lakatos et al., 2020). These documented improvements in LA function supports our results that LAVi enlargement in physically active individuals is benign.

The physiological mechanisms triggering LA remodelling in athletes are the increased hemodynamic stress on the walls due to increased stroke volume and cardiac output during exercise (D'Ascenzi et al., 2018). In contrast, LA enlargement may also reflect the presence of LA dysfunction and increased filling pressures over time (D’Ascenzi et al., 2020, Nagueh et al., 2016). Furthermore, LA enlargement is seen in subjects with AF and valvular disease (Nagueh et al., 2016). However, in our study population only a few participants had AF, and sensitivity analysis revealed that the associations between PA and LAVi were almost unchanged if they were included or not. Also, adjustment for valvular disease and HF did not change the associations between PA and LAVi. This suggests that enlarged LAVi with increasing levels of PA is due to exercise-induced LA remodelling.

### 4.2 LA enlargement and indices of diastolic dysfunction

We observed an association between LAVi enlargement and increased E/e’ratio and TRV among the most inactive participants only. This finding is of importance as increased E/e’ratio is associated with CVD, all-cause mortality and HF (Mitter et al., 2017). Also, elevated TRV indicates elevated LV filling pressure, and is a main criteria for determining LV diastolic dysfunction (Nagueh et al., 2016). However, as mean TRV was 1.8 m/s in the most inactive participants, the insufficiency was well within the border of a normal TRV (Nagueh et al., 2016).

Our results are supported by previous literature which have demonstrated lower E/e’ratio with higher levels of PA (D'Ascenzi et al., 2011), and that older participants reporting ideal levels of PA have lower E/e’ratio compared to those who reported low PA (Hegde et al., 2016). Furthermore, it has been demonstrated that low cardiorespiratory fitness is associated with higher E/e’ratio, compared with higher levels of fitness (Brinker et al., 2014). In contrast, some researchers have reported no association between LAVi and E/e’ratio or TRV (Letnes et al., 2020), or between PA and E/e’ratio (Andersson et al., 2015) in healthy adults or middle-aged. This discrepancy may be explained by a younger and healthier population in these studies. It is suggested that the age-related changes in diastolic function are probably not developed in young cohorts (Hegde et al., 2016).

Furthermore, LA enlargement is an independent risk factor for AF in the general population (Pelliccia et al., 2005). Although it is well documented that moderate levels of PA decreases the risk of AF (Valenzuela et al., 2020), vigorous endurance exercise may be associated with increased risk of AF (D’Ascenzi et al., 2015). However, solid evidence for exercise-induced LA enlargement as a substrate for AF is lacking (D’Ascenzi et al., 2015). Furthermore, in a large study of 1800 competitive athletes, the authors demonstrated frequently LA enlargement, but low prevalence of AF (<1%) (Pelliccia et al., 2005).

### 4.3 Strengths and limitations

The main strength of this study is the combined use of objectively measured PA and echocardiography in a large study population with a wide age span. Our large study population of adult and elderly individuals represents the general population. Self-reported PA is prone to both recall- and social desirability bias (Prince et al., 2008), and is neither able to capture light PA or assess MVPA precisely (The 2018 Physical Activity Guidelines Advisory Committee, 2018). In contrast, device based measured PA provides objective and more valid data across the whole spectrum of PA as well as total accumulated PA throughout the day (Chomistek et al., 2017). The echocardiography was conducted according to current guidelines, and we used the recommended LAVi calculated with the Simpson’s biplane method to assess LA size (Lang et al., 2015).

Potential limitations should be addressed. Firstly, the cross-sectional study design prevents evaluation of longitudinal changes in PA and LA size, or intra-subject LA remodelling, and we cannot establish direction of the association or causation. Secondly, lack of data on LA function prevents extensive evaluation of the association between PA and LA remodelling. Thirdly, lack of data from cardiopulmonary exercise-testing prevents us from evaluating the impact of cardiorespiratory fitness on LA remodelling. Fourthly, although we adjusted for covariates, we cannot exclude the possibility of residual confounding. Fifth, due to few participants with diastolic dysfunction, diastolic function was dichotomized into normal or abnormal. This may have underestimated the influence of diastolic dysfunction on LAVi, as the indeterminate cases (=50% positive variables) were defined as abnormal. Lastly, as the accelerometer fails to register water-based activities and is less accurate in movements involving small trunk motions (e.g. bicycling), the participant’s true PA is probably somewhat underestimated.

In conclusion, increasing level of PA was associated with greater LAVi in the general adult and elderly population < 70 years with normal LV diastolic function, whereas no associations were seen at ages ≥ 70 years or for participants with abnormal diastolic function. Furthermore, LAVi enlargement was only associated with diastolic dysfunction in the most inactive PA-quartile. The beneficial effect of PA on indices of LV diastolic dysfunction was observed from PA-quartile 2, with no additional benefits from increasing PA.

## CRediT authorship contribution statement

**Kim Arne Heitmann:** Conceptualization, Methodology, Validation, Formal analysis, Writing - original draft, Visualization. **Maja-Lisa Løchen:** Conceptualization, Methodology, Validation, Writing - review & editing. **Laila A. Hopstock:** Methodology, Validation, Investigation, Writing - review & editing. **Michael Stylidis:** Conceptualization, Methodology, Validation, Investigation, Writing - review & editing. **Boye Welde:** Validation, Writing - review & editing. **Henrik Schirmer:** Conceptualization, Methodology, Validation, Formal analysis, Writing - review & editing. **Bente Morseth:** Conceptualization, Methodology, Validation, Formal analysis, Investigation, Writing - review & editing, Funding acquisition.

## Declaration of Competing Interest

The authors declare that they have no known competing financial interests or personal relationships that could have appeared to influence the work reported in this paper.
