# Supplementary material for: Cross-sectional associations between accelerometry-measured physical activity, left atrial size, and indices of left ventricular diastolic dysfunction: The Tromsø Study
Source: Prev Med Rep. 2020 Dec 31;21:101290. doi: 10.1016/j.pmedr.2020.101290 (PMC7782323; doi:10.1016/j.pmedr.2020.101290)
Supplement: Supplementary data 1 [file mmc1.docx]

**Cross-sectional associations between accelerometry-measured physical activity, left atrial size, and indices of left ventricular diastolic dysfunction: The Tromsø Study**

**SUPPLEMENTARY MATERIAL**

**Table S1.** Associations between physical activity and left atrial volume index (unstandardized coefficients ±95% CI). The Tromsø Study 2015-16

|  | n | LAVi  mL/m^2^ (SD) | Model 1  β (95% CI) | Model 2  β (95% CI) |
| --- | --- | --- | --- | --- |
| CPM | 1542 | 34.3 (12.3) |  |  |
| PA-quartile 1 | 383 | 35.1 (14.4) | 0.00 (Ref.) | 0.00 (Ref.) |
| PA-quartile 2 | 389 | 33.7 (12.4) | -1.40 (-3.14, 0.34) | -0.07 (-1.81, 1.67) |
| PA-quartile 3 | 384 | 33.5 (12.0) | -1.59 (-3.33, 0.16) | 0.11 (-1.66, 1.88) |
| PA-quartile 4 | 386 | 34.9 (10.2) | -0.19 (-1.93, 1.56) | 2.31 (0.45, 4.17) |
| p-trend |  |  | 0.799 | 0.017 |
| MVPA per 10 min/day | 1542 | 34.3 (12.3) | -0.11 (-0.32, 0.09) | 0.15 (-0.07, 0.37) |
| p-trend |  |  | 0.277 | 0.176 |
| Steps per 1000/day | 1542 | 34.3 (12.3) | -0.17 (-0.38, 0.04) | 0.16 (-0.08, 0.39) |
| p-trend |  |  | 0.103 | 0.184 |

Model 1 was unadjusted. Model 2 was adjusted for age, sex, BMI, systolic blood pressure, diabetes, smoking, and LDL cholesterol.

LAVi: left atrial volume index, SD: standard deviation, CI: confidence interval, Ref.: reference, CPM: counts per minute, PA: physical activity, MVPA: moderate-to-vigorous physical activity, BMI: body mass index, LDL: low-density lipoprotein.

**Table S2.** Test of the significant interaction terms in the multivariable model (p-values). The Tromsø Study 2015-16

|  | n | PA*age^a^ | PA*diastolic function^b^ |
| --- | --- | --- | --- |
| Total PA (CPM) | 1542 | 0.091 | 0.003 |
| MVPA per 10 min/day | 1542 | 0.043 | 0.163 |
| Steps per 1000/day | 1542 | 0.010 | 0.013 |

The model was adjusted for: age, sex, BMI, systolic blood pressure, diabetes, smoking, LDL cholesterol, diastolic function, (PA*age), (PA*diastolic function).

PA: physical activity, MVPA: moderate-to-vigorous physical activity, BMI: body mass index, LDL: low-density lipoprotein.

^a^ Three age groups: 40-54 years, 55-69 years and ≥70 years.

^b^Diastolic function: a) normal if >50% of the variables are below the cut-off values, or b) abnormal if ≥50% of the variables are above the cut-off values.

**Table S3.** Associations between physical activity and left atrial volume index by hypertension (unstandardized coefficients ±95% CI). The Tromsø Study 2015-16

|  | n | LAVi  mL/m^2^ (SD) | Model 1  β (95% CI) | Model 2  β (95% CI) |
| --- | --- | --- | --- | --- |
| **Normal^a^** | | | | |
| CPM | 692 | 33.2 (10.8) |  |  |
| PA-quartile 1 | 108 | 32.8 (13.4) | 0.00 (Ref.) | 0.00 (Ref.) |
| PA-quartile 2 | 163 | 32.9 (11.8) | 0.11 (-2.53, 2.75) | 1.16 (-1.44, 3.76) |
| PA-quartile 3 | 180 | 32.2 (9.6) | -0.61 (-3.20, 1.98) | 0.31 (-2.25, 2.87) |
| PA-quartile 4 | 241 | 34.3 (9.7) | 1.48 (-0.98, 3.94) | 2.73 (0.24, 5.22) |
| p-trend |  |  | 0.205 | 0.040 |
| MVPA per 10 min/day | 692 | 33.2 (10.8) | 0.13 (-0.14, 0.39) | 0.18 (-0.09, 0.45) |
| p-trend |  |  | 0.345 | 0.183 |
| Steps per 1000/day | 692 | 33.2 (10.8) | 0.14 (-0.13, 0.42) | 0.27 (-0.01, 0.55) |
| p-trend |  |  | 0.302 | 0.062 |
| **Hypertension, controlled^b^** | | | | |
| CPM | 246 | 34.9 (13.9) |  |  |
| PA-quartile 1 | 88 | 34.0 (14.3) | 0.00 (Ref.) | 0.00 (Ref.) |
| PA-quartile 2 | 66 | 35.2 (14.9) | 1.23 (-3.27, 5.72) | 3.08 (-1.48, 7.64) |
| PA-quartile 3 | 49 | 35.1 (14.5) | 1.10 (-3.82, 6.02) | 2.22 (-2.73, 7.17) |
| PA-quartile 4 | 43 | 36.0 (10.9) | 1.97 (-3.17, 7.10) | 4.36 (-1.00, 9.72) |
| p-trend |  |  | 0.454 | 0.137 |
| MVPA per 10 min/day | 246 | 34.9 (13.9) | -0.14 (-0.81, 0.53) | 0.30 (-0.42, 1.02) |
| p-trend |  |  | 0.679 | 0.417 |
| Steps per 1000/day | 246 | 34.9 (13.9) | -0.28 (-0.96, 0.40) | 0.13 (-0.65, 0.90) |
| p-trend |  |  | 0.416 | 0.745 |
| **Hypertension^c^** | | | | |
| CPM | 596 | 35.4 (13.2) |  |  |
| PA-quartile 1 | 184 | 37.1 (14.7) | 0.00 (Ref.) | 0.00 (Ref.) |
| PA-quartile 2 | 156 | 33.9 (11.9) | -3.14 (-5.95, -0.34) | -1.92 (-4.77, 0.93) |
| PA-quartile 3 | 155 | 34.5 (13.4) | -2.60 (-5.41, 0.21) | -0.61 (-3.51, 2.29) |
| PA-quartile 4 | 101 | 35.9 (11.2) | -1.18 (-4.37, 2.01) | 1.38 (-2.00, 4.76) |
| p-trend |  |  | 0.316 | 0.458 |
| MVPA per 10 min/day | 596 | 35.4 (13.2) | -0.25 (-0.62, 0.11) | 0.08 (-0.31, 0.48) |
| p-trend |  |  | 0.173 | 0.678 |
| Steps per 1000/day | 596 | 35.4 (13.2) | -0.36 (-0.73, 0.02) | 0.04 (-0.38, 0.46) |
| p-trend |  |  | 0.063 | 0.850 |

Model 1 was unadjusted. Model 2 was adjusted for age, sex, BMI, diabetes, smoking, and LDL cholesterol.

LAVi: left atrial volume index, SD: standard deviation, CI: confidence interval, Ref.: reference, CPM: counts per minute, PA: physical activity, MVPA: moderate-to-vigorous physical activity, BMI: body mass index, LDL: low-density lipoprotein.

^a^Normal: <140 mmHg systolic blood pressure and <90 mmHg diastolic blood pressure and no self-reported use antihypertensives.

^b^Hypertension controlled: <140 mmHg systolic blood pressure and <90 mmHg diastolic blood pressure, and self-reported use of antihypertensives.

^c^Hypertension: systolic blood pressure ≥140 mmHg and/or diastolic blood pressure ≥90 mmHg and no self-reported use antihypertensives.

**Table S4.** Associations between physical activity and left atrial volume index by history of cardiovascular diseases (unstandardized coefficients ±95% CI). The Tromsø Study 2015-16

|  | n | LAVi  mL/m^2^ (SD) | Model 1  β (95% CI) | Model 2  β (95% CI) |
| --- | --- | --- | --- | --- |
| **Non-CVD^a^** | | | | |
| CPM | 1372 | 33.7 (11.8) |  |  |
| PA-quartile 1 | 301 | 34.0 (13.6) | 0.00 (Ref.) | 0.00 (Ref.) |
| PA-quartile 2 | 351 | 33.3 (12.4) | -0.78 (-2.60, 1.04) | 0.36 (-1.47, 2.19) |
| PA-quartile 3 | 351 | 33.0 (11.4) | -1.02 (-2.84, 0.80) | 0.43 (-1.42, 2.28) |
| PA-quartile 4 | 369 | 34.6 (9.9) | 0.56 (-1.24, 2.36) | 2.58 (0.67, 4.50) |
| p-trend |  |  | 0.537 | 0.009 |
| MVPA per 10 min/day | 1372 | 33.7 (11.8) | -0.02 (-0.23, 0.18) | 0.18 (-0.04, 0.40) |
| p-trend |  |  | 0.825 | 0.115 |
| Steps per 1000/day | 1372 | 33.7 (11.8) | -0.09 (-0.30, 0.12) | 0.17 (-0.07, 0.40) |
| p-trend |  |  | 0.406 | 0.166 |
| **CVD^b^** | | | | |
| CPM | 146 | 38.2 (15.4) |  |  |
| PA-quartile 1 | 71 | 38.6 (16.6) | 0.00 (Ref.) | 0.00 (Ref.) |
| PA-quartile 2 | 33 | 37.8 (11.7) | -0.89 (-7.38, 5.60) | 0.55 (-6.10, 7.20) |
| PA-quartile 3 | 27 | 36.6 (16.9) | -2.06 (-9.02, 4.91) | 0.70 (-6.61, 8.00) |
| PA-quartile 4 | 15 | 40.4 (15.5) | 1.79 (-6.96, 10.54) | 5.86 (-3.19, 14.90) |
| p-trend |  |  | 0.970 | 0.310 |
| MVPA per 10 min/day | 146 | 38.2 (15.4) | -0.17 (-1.22, 0.88) | 0.42 (-0.71, 1.56) |
| p-trend |  |  | 0.750 | 0.461 |
| Steps per 1000/day | 146 | 38.2 (15.4) | -0.03 (-1.04, 0.99) | 0.51 (-0.62, 1.63) |
| p-trend |  |  | 0.958 | 0.372 |

Model 1 was unadjusted. Model 2 was adjusted for age, sex, BMI, systolic blood pressure, diabetes, smoking, and LDL cholesterol.

LAVi: left atrial volume index, SD: standard deviation, CI: confidence interval, CVD: cardiovascular diseases, Ref.: reference, CPM: counts per minute, PA: physical activity, MVPA: moderate-to-vigorous physical activity, BMI: body mass index, LDL: low-density lipoprotein.

^a^Non-CVD = No self-reported myocardial infarction or stroke

^b^CVD = Self-reported myocardial infarction and/or stroke

**Table S5.** Associations between physical activity and left atrial volume index by left ventricular mass index (unstandardized coefficients ±95% CI). The Tromsø Study 2015-16

|  | n | LAVi  mL/m^2^ (SD) | Model 1  β (95% CI) | Model 2  β (95% CI) |
| --- | --- | --- | --- | --- |
| **Normal (≤50 g/m^2.7^)** | | | | |
| CPM | 1090 | 32.7 (10.6) |  |  |
| PA-quartile 1 | 238 | 32.4 (11.5) | 0.00 (Ref.) | 0.00 (Ref.) |
| PA-quartile 2 | 269 | 32.5 (11.5) | 0.16 (-1.69, 2.00) | 1.14 (-0.73, 3.01) |
| PA-quartile 3 | 284 | 32.3 (10.8) | -0.05 (-1.87, 1.77) | 1.27 (-0.60, 3.14) |
| PA-quartile 4 | 299 | 33.3 (8.4) | 0.92 (-0.88, 2.72) | 2.57 (0.61, 4.53) |
| p-trend |  |  | 0.326 | 0.013 |
| MVPA per 10 min/day | 1090 | 32.7 (10.6) | -0.00 (-0.21, 0.20) | 0.15 (-0.07, 0.37) |
| p-trend |  |  | 0.983 | 0.170 |
| Steps per 1000/day | 1090 | 32.7 (10.6) | 0.06 (-0.15, 0.27) | 0.25 (0.01, 0.48) |
| p-trend |  |  | 0.552 | 0.039 |
| **Hypertrophy (>50 g/m^2.7^)** | | | | |
| CPM | 434 | 38.3 (15.4) |  |  |
| PA-quartile 1 | 123 | 40.4 (18.1) | 0.00 (Ref.) | 0.00 (Ref.) |
| PA-quartile 2 | 116 | 36.8 (14.0) | -3.53 (-7.42, 0.37) | -2.42 (-6.33, 1.51) |
| PA-quartile 3 | 104 | 36.2 (14.5) | -4.17 (-8.18, -0.17) | -2.65 (-6.76, 1.47) |
| PA-quartile 4 | 91 | 39.8 (13.6) | -0.62 (-4.78, 3.54) | 1.12 (-3.22, 5.46) |
| p-trend |  |  | 0.727 | 0.587 |
| MVPA per 10 min/day | 434 | 38.3 (15.4) | -0.15 (-0.67, 0.36) | 0.04 (-0.51, 0.59) |
| p-trend |  |  | 0.562 | 0.887 |
| Steps per 1000/day | 434 | 38.3 (15.4) | -0.36 (-0.89, 0.17) | -0.09 (-0.68, 0.50) |
| p-trend |  |  | 0.187 | 0.768 |

Model 1 was unadjusted. Model 2 was adjusted for age, sex, BMI, systolic blood pressure, diabetes, smoking, and LDL cholesterol.

LAVi: left atrial volume index, SD: standard deviation, CI: confidence interval, Ref.: reference, CPM: counts per minute, PA: physical activity, MVPA: moderate-to-vigorous physical activity, BMI: body mass index, LDL: low-density lipoprotein.

**Table S6.** Associations between physical activity and left atrial volume index by sex (unstandardized coefficients ±95% CI). The Tromsø Study 2015-16

|  | n | LAVi  mL/m^2^ (SD) | Model 1  β (95% CI) | Model 2  β (95% CI) |
| --- | --- | --- | --- | --- |
| **Male** | | | | |
| CPM | 745 | 34.9 (13.5) |  |  |
| PA-quartile 1 | 205 | 36.3 (15.9) | 0.00 (Ref.) | 0.00 (Ref.) |
| PA-quartile 2 | 187 | 33.0 (11.6) | -3.34 (-6.01, -0.68) | -1.19 (-3.87, 1.49) |
| PA-quartile 3 | 182 | 34.1 (13.6) | -2.23 (-4.91, 0.46) | 0.17 (-2.56, 2.90) |
| PA-quartile 4 | 171 | 36.1 (11.7) | -0.16 (-2.88, 2.57) | 2.79 (-0.09, 5.67) |
| p-trend |  |  | 0.988 | 0.041 |
| MVPA per 10 min/day | 745 | 34.9 (13.5) | -0.08 (-0.39, 0.23) | 0.23 (-0.10, 0.56) |
| p-trend |  |  | 0.607 | 0.178 |
| Steps per 1000/day | 745 | 34.9 (13.5) | -0.25 (-0.59, 0.09) | 0.19 (-0.19, 0.57) |
| p-trend |  |  | 0.146 | 0.325 |
| **Female** | | | | |
| CPM | 797 | 33.7 (11.1) |  |  |
| PA-quartile 1 | 178 | 33.6 (12.3) | 0.00 (Ref.) | 0.00 (Ref.) |
| PA-quartile 2 | 202 | 34.3 (13.1) | 0.67 (-1.58, 2.93) | 0.99 (-1.25, 3.24) |
| PA-quartile 3 | 202 | 32.9 (10.2) | -0.70 (-2.95, 1.55) | -0.02 (-2.29, 2.25) |
| PA-quartile 4 | 215 | 33.9 (8.8) | 0.23 (-1.99, 2.45) | 1.81 (-0.56, 4.18) |
| p-trend |  |  | 0.847 | 0.258 |
| MVPA per 10 min/day | 797 | 33.7 (11.1) | -0.16 (-0.42, 0.10) | 0.05 (-0.23, 0.34) |
| p-trend |  |  | 0.231 | 0.709 |
| Steps per 1000/day | 797 | 33.7 (11.1) | -0.09 (-0.35, 0.16) | 0.13 (-0.15, 0.41) |
| p-trend |  |  | 0.466 | 0.368 |

Model 1 was unadjusted. Model 2 was adjusted for age, BMI, systolic blood pressure, diabetes, smoking, and LDL cholesterol.

LAVi: left atrial volume index, SD: standard deviation, CI: confidence interval, Ref.: reference, CPM: counts per minute, PA: physical activity, MVPA: moderate-to-vigorous physical activity, BMI: body mass index, LDL: low-density lipoprotein.

**Table S7.** Associations between physical activity and left atrial volume index by body mass index (unstandardized coefficients ±95% CI). The Tromsø Study 2015-16

|  | n | LAVi  mL/m^2^ (SD) | Model 1  β (95% CI) | Model 2  β (95% CI) |
| --- | --- | --- | --- | --- |
| **BMI <25** | | | | |
| CPM | 518 | 34.7 (12.9) |  |  |
| PA-quartile 1 | 104 | 35.1 (15.4) | 0.00 (Ref.) | 0.00 (Ref.) |
| PA-quartile 2 | 112 | 35.5 (15.2) | 0.40 (-3.05, 3.85) | 1.70 (-1.71, 5.11) |
| PA-quartile 3 | 132 | 33.6 (11.2) | -1.56 (-4.88, 1.76) | -0.07 (-3.36, 3.23) |
| PA-quartile 4 | 170 | 34.9 (10.5) | -0.22 (-3.37, 2.93) | 2.41 (-0.85, 5.66) |
| p-trend |  |  | 0.671 | 0.270 |
| MVPA per 10 min/day | 518 | 34.7 (12.9) | -0.01 (-0.36, 0.34) | 0.26 (-0.11, 0.62) |
| p-trend |  |  | 0.961 | 0.167 |
| Steps per 1000/day | 518 | 34.7 (12.9) | -0.13 (-0.48, 0.21) | 0.19 (-0.18, 0.56) |
| p-trend |  |  | 0.445 | 0.309 |
| **BMI 25-29.9** | | | | |
| CPM | 703 | 34.0 (10.7) |  |  |
| PA-quartile 1 | 165 | 34.8 (12.7) | 0.00 (Ref.) | 0.00 (Ref.) |
| PA-quartile 2 | 190 | 32.6 (10.3) | -2.18 (-4.41, 0.05) | -1.27 (-3.52, 0.98) |
| PA-quartile 3 | 180 | 34.1 (11.1) | -0.73 (-2.99, 1.53) | 0.57 (-1.72, 2.86) |
| PA-quartile 4 | 168 | 34.5 (8.3) | -0.36 (-2.66, 1.94) | 1.39 (-1.06, 3.83) |
| p-trend |  |  | 0.870 | 0.096 |
| MVPA per 10 min/day | 703 | 34.0 (10.7) | -0.15 (-0.41, 0.12) | 0.06 (-0.23, 0.34) |
| p-trend |  |  | 0.284 | 0.694 |
| Steps per 1000/day | 703 | 34.0 (10.7) | -0.17 (-0.45, 0.12) | 0.09 (-0.22, 0.41) |
| p-trend |  |  | 0.259 | 0.561 |
| **BMI ≥30** | | | | |
| CPM | 321 | 34.2 (14.6) |  |  |
| PA-quartile 1 | 114 | 35.4 (15.7) | 0.00 (Ref.) | 0.00 (Ref.) |
| PA-quartile 2 | 87 | 33.5 (12.4) | -1.87 (-5.96, 2.21) | 0.38 (-3.72, 4.48) |
| PA-quartile 3 | 72 | 31.8 (15.1) | -3.59 (-7.91, 0.73) | -0.14 (-4.61, 4.33) |
| PA-quartile 4 | 48 | 36.2 (14.6) | 0.87 (-4.06, 5.81) | 4.61 (-0.50, 9.72) |
| p-trend |  |  | 0.693 | 0.169 |
| MVPA per 10 min/day | 321 | 34.2 (14.6) | -0.34 (-0.93, 0.26) | 0.17 (-0.47, 0.80) |
| p-trend |  |  | 0.267 | 0.609 |
| Steps per 1000/day | 321 | 34.2 (14.6) | -0.46 (-1.08, 0.15) | 0.15 (-0.52, 0.82) |
| p-trend |  |  | 0.142 | 0.662 |

Model 1 was unadjusted. Model 2 was adjusted for age, sex, systolic blood pressure, diabetes, smoking, and LDL cholesterol.

LAVi: left atrial volume index, SD: standard deviation, CI: confidence interval, Ref.: reference, CPM: counts per minute, PA: physical activity, MVPA: moderate-to-vigorous physical activity, BMI: body mass index, LDL: low-density lipoprotein.

**Table S8.** Associations between physical activity and left atrial volume index by age (unstandardized coefficients ±95% CI). The Tromsø Study 2015-16

|  | n | LAVi  mL/m^2^ (SD) | Model 3  β (95% CI) |
| --- | --- | --- | --- |
| **Age 40-54** | | | |
| CPM | 258 | 32.2 (9.1) |  |
| PA-quartile 1 | 23 | 30.1 (6.4) | 0.00 (Ref.) |
| PA-quartile 2 | 59 | 31.3 (11.3) | 1.17 (-3.09, 5.42) |
| PA-quartile 3 | 69 | 29.5 (8.0) | -0.96 (-5.17, 3.25) |
| PA-quartile 4 | 107 | 34.8 (8.2) | 4.03 (0.01, 8.05) |
| p-trend |  |  | 0.017 |
| MVPA per 10 min/day | 258 | 32.2 (9.1) | 0.32 (-0.05, 0.69) |
| p-trend |  |  | 0.089 |
| Steps per 1000/day | 258 | 32.2 (9.1) | 0.54 (0.12, 0.97) |
| p-trend |  |  | 0.012 |
| **Age 55-69** | | | |
| CPM | 633 | 32.6 (10.8) |  |
| PA-quartile 1 | 107 | 31.4 (12.6) | 0.00 (Ref.) |
| PA-quartile 2 | 159 | 31.2 (9.3) | 1.04 (-1.43, 3.51) |
| PA-quartile 3 | 183 | 33.2 (10.7) | 3.06 (0.64, 5.48) |
| PA-quartile 4 | 184 | 33.9 (10.7) | 4.00 (1.56, 6.45) |
| p-trend |  |  | <0.001 |
| MVPA per 10 min/day | 633 | 32.6 (10.8) | 0.39 (0.12, 0.65) |
| p-trend |  |  | 0.004 |
| Steps per 1000/day | 633 | 32.6 (10.8) | 0.34 (0.05, 0.62) |
| p-trend |  |  | 0.021 |
| **Age ≥70** | | | |
| CPM | 446 | 37.4 (14.0) |  |
| PA-quartile 1 | 187 | 37.6 (15.3) | 0.00 (Ref.) |
| PA-quartile 2 | 115 | 38.2 (14.8) | 0.60 (-2.59, 3.79) |
| PA-quartile 3 | 88 | 35.7 (11.6) | -0.50 (-4.03, 3.03) |
| PA-quartile 4 | 56 | 38.1 (11.3) | 1.31 (-2.85, 5.48) |
| p-trend |  |  | 0.742 |
| MVPA per 10 min/day | 446 | 37.4 (14.0) | -0.16 (-0.68, 0.35) |
| p-trend |  |  | 0.537 |
| Steps per 1000/day | 446 | 37.4 (14.0) | -0.11 (-0.64, 0.42) |
| p-trend |  |  | 0.684 |

Model 3 was adjusted for sex, BMI, systolic blood pressure, diabetes, smoking, LDL cholesterol, myocardial infarction, heart failure, mitral regurgitation, mitral stenosis, aortic regurgitation, and mean aortic valve flow.

LAVi: left atrial volume index, Ref.: reference, CPM: counts per minute, PA: physical activity, MVPA: moderate-to-vigorous physical activity, BMI: body mass index, LDL: low-density lipoprotein.

**Table S9.** Associations between physical activity and left atrial volume index by left ventricular diastolic function (unstandardized coefficients ±95% CI). The Tromsø Study 2015-16

|  | n | LAVi  mL/m^2^ (SD) | Model 3  β (95% CI) |
| --- | --- | --- | --- |
| **Normal diastolic function^a^** | | | |
| CPM | 972 | 30.9 (9.1) |  |
| PA-quartile 1 | 201 | 29.9 (9.2) | 0.00 (Ref.) |
| PA-quartile 2 | 238 | 30.1 (9.9) | 0.43 (-1.29, 2.16) |
| PA-quartile 3 | 261 | 30.6 (9.0) | 1.06 (-0.65, 2.78) |
| PA-quartile 4 | 272 | 32.7 (8.3) | 3.18 (1.40, 4.69) |
| p-value for trend |  |  | <0.001 |
| MVPA per 10 min/day | 972 | 30.9 (9.1) | 0.27 (0.06, 0.47) |
| p-trend |  |  | 0.010 |
| Steps per 1000/day | 972 | 30.9 (9.1) | 0.29 (0.07, 0.51) |
| p-trend |  |  | 0.010 |
| **Abnormal diastolic function^b^** | | | |
| CPM | 365 | 42.8 (13.9) |  |
| PA-quartile 1 | 116 | 43.7 (17.0) | 0.00 (Ref.) |
| PA-quartile 2 | 95 | 42.6 (13.0) | -0.47 (-4.13, 3.20) |
| PA-quartile 3 | 79 | 41.5 (11.4) | -0.74 (-4.75, 3.27) |
| PA-quartile 4 | 75 | 42.9 (12.2) | -1.10 (-5.22, 3.03) |
| p-trend |  |  | 0.589 |
| MVPA per 10 min/day | 365 | 42.8 (13.9) | -0.07 (-0.57, 0.42) |
| p-trend |  |  | 0.778 |
| Steps per 1000/day | 365 | 42.8 (13.9) | -0.25 (-0.77, 0.27) |
| p-trend |  |  | 0.348 |

Model 3 was adjusted for age, sex, BMI, systolic blood pressure, diabetes, smoking, LDL cholesterol, myocardial infarction, heart failure, mitral regurgitation, mitral stenosis, aortic regurgitation, and mean aortic valve flow.

LAVi: left atrial volume index, Ref.: reference, CPM: counts per minute, PA: physical activity, MVPA: moderate-to-vigorous physical activity, BMI: body mass index, LDL: low-density lipoprotein.

^a^Normal diastolic function = <50% positive variables.

^b^Abnormal diastolic function = ≥50% positive variables.

**Table S10.** Association between enlarged left atrial volume index (≥34 mL/m^2^) and indices of diastolic dysfunction, stratified by PA-quartiles (unstandardized coefficients ±95% CI). The Tromsø Study 2015-16

|  | n | Adjusted mean (SE) | Model 1  β (95% CI) | Model 2  β (95% CI) | Model 1  p-trend | Model 2  p-trend |
| --- | --- | --- | --- | --- | --- | --- |
| **Tricuspid regurgitation velocity (m/s)^a^** | | | | | | |
| PA-quartile 1 | 162 | 1.8 (0.1) | 0.02 (0.01-0.03) | 0.02 (0.01-0.03) | <0.001 | <0.001 |
| PA-quartile 2 | 153 | 1.4 (0.2) | 0.01 (0.00-0.02) | 0.01 (-0.01-0.02) | 0.044 | 0.335 |
| PA-quartile 3 | 149 | 1.6 (0.1) | 0.00 (-0.01-0.01) | 0.01 (-0.01-0.01) | 0.796 | 0.815 |
| PA-quartile 4 | 182 | 1.5 (0.2) | 0.01 (-0.01-0.02) | 0.01 (-0.01-0.02) | 0.344 | 0.315 |
| **E/e’ ratio^a^** | | | | | | |
| PA-quartile 1 | 158 | 13.1 (1.0) | 0.26 (0.17-0.35) | 0.24 (0.15-0.33) | <0.001 | <0.001 |
| PA-quartile 2 | 148 | 9.0 (1.5) | 0.04 (-0.07-0.14) | -0.00 (-0.11-0.10) | 0.475 | 0.943 |
| PA-quartile 3 | 147 | 11.0 (0.8) | 0.06 (0.00-0.12) | 0.04 (-0.02-0.10) | 0.044 | 0.163 |
| PA-quartile 4 | 180 | 12.1 (0.7) | -0.01 (-0.07-0.05) | -0.02 (-0.07-0.03) | 0.765 | 0.458 |

Model 1 was unadjusted. Model 2 was adjusted for PA level, sex, BMI, systolic blood pressure, diabetes, smoking, and LDL cholesterol.

PA: physical activity, SE: standard error, CI: confidence interval, MVPA: moderate-to-vigorous physical activity, BMI: body mass index, LDL: low-density lipoprotein.
